# NR2A contributes to genesis and propagation of cortical spreading depression in rats

**DOI:** 10.1038/srep23576

**Published:** 2016-03-22

**Authors:** Fan Bu, Ruoxing Du, Yi Li, John P Quinn, Minyan Wang

**Affiliations:** 1 Department of Biological Sciences, Xi’an Jiaotong-Liverpool University, Suzhou, 215123, China; 2 Centre for Neuroscience, Xi’an Jiaotong-Liverpool University, Suzhou, 215123, China; 3 Department of Applied Chemistry, Xi’an Jiaotong-Liverpool University, Suzhou, 215123, China; 4 Department of Molecular and Clinical Pharmacology, Institute of Translational Medicine, University of Liverpool, Liverpool, L69 7ZB, UK

## Abstract

Cortical spreading depression (CSD) is a transient propagating excitation of synaptic activity followed by depression, which is implicated in migraine. Increasing evidence points to an essential role of NR2A-containing NMDA receptors in CSD propagation *in vitro*; however, whether these receptors mediate CSD genesis *in vivo* requires clarification and the role of NR2A on CSD propagation is still under debate. Using *in vivo* CSD in rats with electrophysiology and *in vitro* CSD in chick retina with intrinsic optical imaging, we addressed the role of NR2A in CSD. We demonstrated that NVP-AAM077, a potent antagonist for NR2A-containing receptors, perfused through microdialysis probes, markedly reduced cortex susceptibility to CSD, but also reduced magnitude of CSD genesis in rats. Additionally, NVP-AAM077 at 0.3 nmol perfused into the contralateral ventricle, considerably suppressed the magnitude of CSD propagation wave and propagation rate in rats. This reduction in CSD propagation was also observed with TCN-201, a negative allosteric modulator selective for NR2A, at 3 μM, in the chick retina. Our data provides strong evidence that NR2A subunit contributes to CSD genesis and propagation, suggesting drugs selectively antagonizing NR2A-containing receptors might constitute a highly specific strategy treating CSD associated migraine with a likely better safety profile.

Cortical Spreading Depression (CSD) is a temporary excitation of synaptic activity^1^,^2^, followed by depression that propagates slowly across the cerebral cortex. CSD is implicated in pathophysiological mechanisms of migraine aura^3^,^4^,^5^ and may lead to migraine headache via central modulation involving the activation of neuronal PANX1 protein^6^. CSD is accompanied by a rapid disruption of ionic homeostasis, release of neurotransmitters, neuropeptides and changes in cerebral blood flow; however, the molecular pathways that mediate the initial phase of CSD and its role in the development of migraine are still poorly understood.

N-methyl-D-aspartate (NMDA) receptor activation is known to contribute to CSD genesis and propagation as the non-competitive inhibitor, MK801, appears to be the most effective anti-CSD compound in rats^7^,^8^. However, this drug is perceived as an unlikely anti-CSD candidate for migraine aura treatment because of unacceptable side effects resulting from the blockade of normal neuronal function^9^,^10^. The NMDA receptor is composed of two mandatory NR1 subunits and at least two NR2 (A-D) or a NR3 (A-B) subunit^11^. NR2A typically mediate synaptic transmission whereas NR2B-containing receptors are located extrasynaptically^12^,^13^ on astrocytes^14^. Increasing evidence has demonstrated that these two major receptor subunits, NR2A^15^ and NR2B^8^,^16^ play critical roles in CSD modulation, thus drugs selectively antagonizing these subunits have been proposed as potential candidates for migraine therapy, having better efficacy and a better safety profile^8^,^15^.

However, whether NR2A-containing receptors modulate susceptibility of the cortex to CSD is unknown. Additionally, whether NR2A-containing receptors contribute to CSD propagation is still a matter of debate^15^,^16^. We have demonstrated that inhibition of NR2A-containing receptors by NVP-AAM077, an antagonist that is preferably selective for NR2A at the concentration applied, resulted in suppression of CSD propagation in chick retina, a tissue which lacks blood vessels^15^, suggesting involvement of neuronal and glial activity. However, using blood oxygen level dependent (BOLD) fMRI approach, NR2A inhibition under TCN-201, a negative allosteric modulator selective for NR2A-containing NMDA receptors, application did not alter CSD propagation features in rats^16^. Therefore, the role of NR2A in CSD propagation requires further clarification *in vivo*.

The primary aim of this study was to explore the role of NR2A-containing receptors in CSD genesis and propagation in rats by investigating effects of NVP-AAM077. The involvement of NR2A in CSD propagation was subsequently confirmed using another pharmacological tool TCN-201 at concentrations selective for binding to NR2A in chick retina.

## Results

### Validation of microdialysis-based CSD model under isoflurane anaesthesia

**Figure 1:**
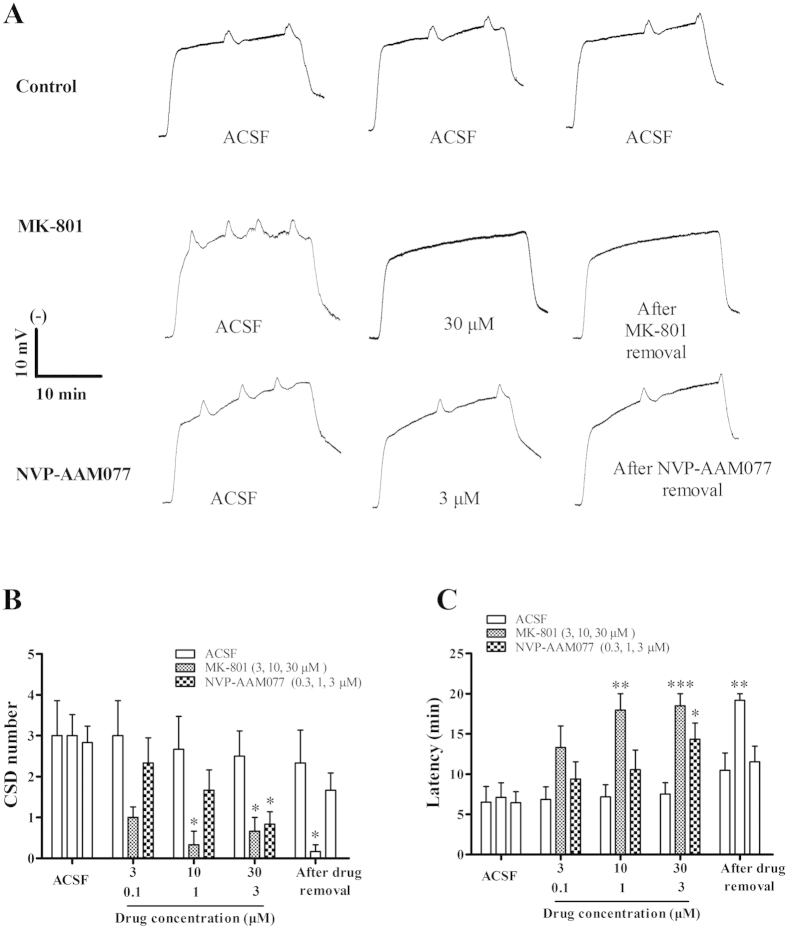
Representative CSD traces (**A**) and effects of MK-801 and NVP-AAM077 on tissue susceptibility to CSD induced by high K^+^ perfused through the microdialysis probe. Three groups including ACSF solution (*n* = 6) as control, MK-801 (*n* = 6) and NVP-AAM077 (*n* = 6) were considered. All the values given are means ± SEM. One-way ANOVA test was used for statistics of multiple comparisons, with subsequent Bonferonni test for significance between control and drug treatment groups (*p < 0.05, **p ≤ 0.01, ***p ≤ 0.001). Paired *t*-test used to test whether tissue was restored after drug removal between the 4^th^ and 5^th^ CSD episodes.

**Figure 2:**
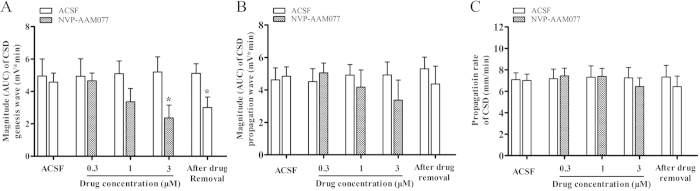
Effects of NVP-AAM077 perfused through the microdialysis probe on the magnitude of both CSD genesis (**A**), propagation waves (**B**) and propagation rate (**C**). All the values shown are means ± SEM. Unpaired t-test was used for statistical analysis for significance between the drug (*n* = 6) and control group (*n* = 6) (*p < 0.05). Paired *t*-test used to test whether tissue was restored after drug removal between the 4^th^ and 5^th^ CSD episodes.

At the CSD genesis site of the control group (ACSF, *n* = 6), the average number, latency and the magnitude of the 1^st^ CSD episode was 3.0 ± 0.86 (Fig. 1B), 6.53 ± 1.93 minutes (Fig. 1C) and 4.95 ± 1.06 mV × minute (Fig. 2A, *n* = 6), in respective order. There was no significant difference in any of these parameters over the five repeated CSD episodes (Figs 1B,C and 2A). To validate the CSD model under anesthesia with isoflurane, the non-competitive NMDA receptor antagonist, MK801, with known anti-CSD effect^7^,^15^, was examined through the microdialysis probe. MK-801 suppressed CSD genesis in a concentration dependent manner (Fig. 1, *n* = 6). MK801 at 30 μM significantly reduced CSD number (0.67 ± 0.33) compared to the control group (2.5 ± 0.62) (One-Way ANOVA, *p* = 0.019, F = 5.236) (Fig. 1B). At this concentration, CSD latency increased 2.5-folds by MK801 compared to the control group (One-Way ANOVA, *p* = 0.001, F = 9.88) (Fig. 1C) and CSD waves were completely abolished in five out of six experiments during the 20-minute recording period (Fig. 1A). This inhibitory effect of MK801 on CSD was persistent after the drug removal (*i.e*. in the 5^th^ CSD episode, Fig. 1A–C).

### Suppression of CSD genesis by NVP-AAM077 applied through microdialysis

To investigate whether NVP-AAM077 alters CSD genesis, the microdialysis-based CSD model was used for local application of both K^+^-medium and the drug. In the control group, CSD number in the 2^nd^, 3^rd^ and 4^th^ episodes was 3.0 ± 0.86, 2.67 ± 0.8, and 2.53 ± 0.62 respectively (Fig. 1A,B, *n* = 6). NVP-AAM077 at 0.3, 1 and 3 μM perfused through microdialysis probes concentration dependently reduced CSD number to 2.33 ± 0.61, 1.67 ± 0.49 and 0.83 ± 0.31 in respectively order (Fig. 1A,B, *n* = 6). At the highest concentration applied, the reduction in CSD number reached significance (*p* = 0.036, F = 5.236, *n* = 6, Fig. 1B).

In the control group, CSD latency in the 2^nd^, 3^rd^ and 4^th^ CSD episodes was 6.85 ± 1.57, 7.18 ± 1.52, and 7.52 ± 1.41 minutes, in respective order (Fig. 1A). Conversely to CSD number, NVP-AAM077 all three concentrations applied prolonged CSD latency to 9.39 ± 2.15, 10.56 ± 2.43 and 13.94 ± 2.23 minutes in the corresponding CSD episodes. The drug at 3 μM perfused through the microdialysis probe significantly prolonged CSD latency when compared with the corresponding control (*p* = 0.041, F = 9.88, Fig. 1A,C, *n* = 6). There was no significant effect of NVP-AAM077 on CSD number and latency after the drug removal.

In the control group, CSD magnitude in the 2^nd^, 3^rd^ and 4^th^ CSD episodes was 4.94 ± 1.08, 5.45 ± 0.86, and 5.20 ± 0.94 mV × minute, in respective order. NVP-AAM077 also significantly reduced CSD magnitude in a concentration dependent manner (Fig. 2A, *n* = 6). NVP-AAM077 at 0.3, 1 and 3 μM reduced the magnitude to 4.66 ± 0.48, 3.36 ± 0.82 and 2.36 ± 0.79 (t-test, *p* = 0.043, F = 1.411) mV × minute, in respective order. This inhibitory effect slightly recovered to 3.00 ± 0.67 mV × minute after the drug removal, which, however, did not reach significance when compared with that of the 4^th^ episode in NVP-AAM077 (3 μM) group.

### Suppression of CSD propagation by NVP-AAM077 perfused into contralateral ventricle but not via the microdialysis probe

Distinct from that of NVP-AAM077 on CSD genesis, perfusion of NVP-AAM077 through the microdialysis probe was unable to alter the magnitude of CSD propagation wave at the propagation site (Fig. 2B, *n* = 6). This route of delivery did not alter the velocity of CSD at all concentrations tested when compared with the control group (Fig. 2C, *n* = 6).

**Figure 3:**
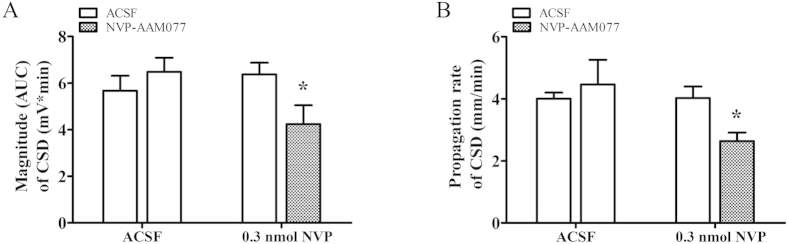
Effects of NVP-AAM077 perfused into the left lateral ventricle on the magnitude of CSD propagation (**A**) and propagation rate (**B**). All the values shown are means ± SEM. Unpaired t-test was used for statistical analysis for significance between the drug (*n* = 6) and control group (*n* = 6) (*p < 0.05).

To allow NVP-AAM077 diffusing to distant cortical layer for investigating CSD propagation under NR2A inhibition, perfusion of the drug into contralateral ventricle was performed (see methods). In the control group, the magnitude of CSD propagation wave at the anterior recording site (0.4 mm to bregma) and propagation rate was 5.67 ± 0.64 mV × minute, and 4.0 ± 0.2 mm/minute respectively (Fig. 3A,B, *n* = 6). There was no change of these parameters throughout the experiment. In contrast to that perfused via microdialysis probe, 0.3 nmol of NVP-AAM077 perfused into the contralateral ventricle significantly suppressed both the magnitude of CSD propagation wave (Fig. 3A, t-test, *p* = 0.044, t = 2.302) and propagation rate (Fig. 3B, t-test, *p* = 0.013, t = 3.008). In the drug group, the magnitude of CSD propagation wave was reduced to 4.20 ± 0.80 mV × minute compared to 6.37 ± 0.50 mV × minute in the control group (Fig. 3A, *n* = 6). Similarly, the propagation rate slowed down to 2.64 ± 0.27 mm/minutes compared to 4.02 ± 0.37 mm/minutes in the control group (Fig. 3B, *n* = 6) when measured between the two recording electrode implantation sites.

### Suppression of CSD by TCN-201 in chick retina

To confirm NR2A-containing NMDA receptors are involved in CSD propagation, we examined the effect of TCN-201 that was previously reported to have better selectivity for NR2A than NVP-AAM077^17^ in CSD propagation in chick retina. Effect of NVP-AAM077 on CSD propagation was also designed for comparison.

**Figure 4:**
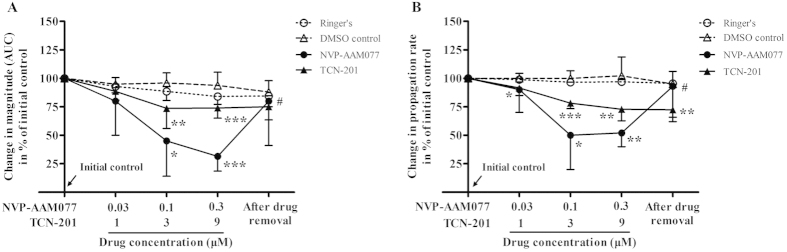
Effects of NVP-AAM077 (*n* = 6) and TCN-201 (*n* = 6) on the magnitude (**A**) and propagation rate (**B**) of CSD propagation induced by K^+^ in the chick retina. Data was plotted as percentage of their initial levels and indicated as mean ± SD. Kruskal–Wallis test was used for statistical analysis of multiple comparisons, with subsequent Dunns test for significance (*p < 0.05, **p ≤ 0.01, ***p ≤ 0.001, TCN-201 *vs.* DMSO control (*n* = 8) or NVP-AAM077 *vs.* Ringer’s control (*n* = 6)). Wilcoxon matched pairs test was used to test whether tissue was restored after drug removal (^#^*p* < 0.05, 0.3 μM NVP-AAM077 *vs.* drug removal).

In the Ringer’s group, there was no significant change in the magnitude and propagation rate over repeated CSD episodes throughout the experiment (Fig. 4, *n* = 6). Compared with the Ringer’s group, both CSD magnitude and propagation rate were markedly suppressed by NVP-AAM077 in a concentration-dependent manner (Fig. 4A,B, *n* = 6), which is in consistent with that reported previously^15^. At the maximum concentration tested (0.3 μM), the magnitude and propagation rate of CSD was reduced to 31.5% (Kruskal–Wallis test, *p* = 0.0001, H = 20.47) and 52% (Kruskal–Wallis test, *p* = 0.003, H = 18.97) of initial level respectively. This inhibitory effect significantly recovered to 80.2% (Wilcoxon matched pairs test, *p* = 0.03, W = 21) and 88.2% (Wilcoxon matched pairs test, *p* = 0.032, W = 20.56) of initial level after drug removal (high concentration *vs* drug removal).

Similar to the result of NVP-AAM077, but to a lesser extent, TCN-201 at both the medium and highest concentrations applied, markedly reduced the magnitude and propagation rate (Fig. 4A,B, *n* = 6), when compared with their respective DMSO group (*n* = 8). At 9 μM, the magnitude and propagation rate of RSD was reduced to 73.8% (Kruskal–Wallis test, *p* = 0.0001, H = 20.47) and 72.8% (Kruskal–Wallis test, *p* = 0.003, H = 18.97) of the initial level respectively. This inhibitory effect did not recover after drug removal (Fig. 4).

## Discussion

Our *in vivo* data reveals, for the first time, a critical role of NR2A-containing receptors in CSD genesis as demonstrated by that NVP-AAM077 perfused via microdialysis probes not only reduced cortex susceptibility to K^+^-induced CSD but also suppressed the magnitude of CSD elicitation in rats (Figs 1 and 2). Additionally, the electrophysiology study with NVP-AAM077 in rats (Fig. 3) and the application of TCN-201 in chick retina (Fig. 4) extended our previously findings on NR2A-containing receptor activation contributing to CSD propagation^15^.

### Validation of microdialysis-based CSD model under isoflurane anesthesia

Several studies reported that inhalational anaesthetics suppress CSD^18^,^19^. In this study, we still considered isoflurane anesthesia, but not pentobarbitone, which has the least action on CSD^20^, for studying genesis and propagation of CSD for the following reasons: (1) our *in vivo* experiment lasted almost 7 hours, whereas barbiturates usually only maintain anesthesia from several minutes to 4 hours^21^, which did not meet our requirement; (2) Isoflurane has been widely used in other studies for studying CSD^8^,^16^,^22^,^23^; indeed, isoflurane provided a stable and consistent depth of anesthesia throughout the experiment and the concentration could be adjusted according to surgical and CSD recording procedures under study; whereas barbiturates generally result in uneven levels of anesthesia^21^; (3) Under isoflurane anaesthesia, the profound inhibitory effects of MK801 on CSD magnitude and susceptibility of the cortex to CSD (Fig. 1) were consistent with those reported previously^24^,^25^; (4) The fact that neither genesis nor propagation of CSD was altered in the control group yet was suppressed by NVP-AAM077, suggests that the suppression of CSD was due to NR2A inhibition, rather than to isoflurane. Collectively, the above evidence validates that the *in vivo* study under isoflurane anaesthesia is feasible and valid for assessing CSD genesis and propagation for NMDA receptor pharmacology.

### NR2A-containing receptors mediate both CSD genesis and propagation

NVP-AAM077 markedly suppressed K^+^-induced CSD number and increased CSD latency when perfused via microdialysis probes at 0.3 μM (Fig. 1). The reduced cortex susceptibility to CSD under NR2A inhibition extended our previous finding on the crucial role NR2A-containing receptors in mediating CSD propagation^15^.

**Figure 5:**
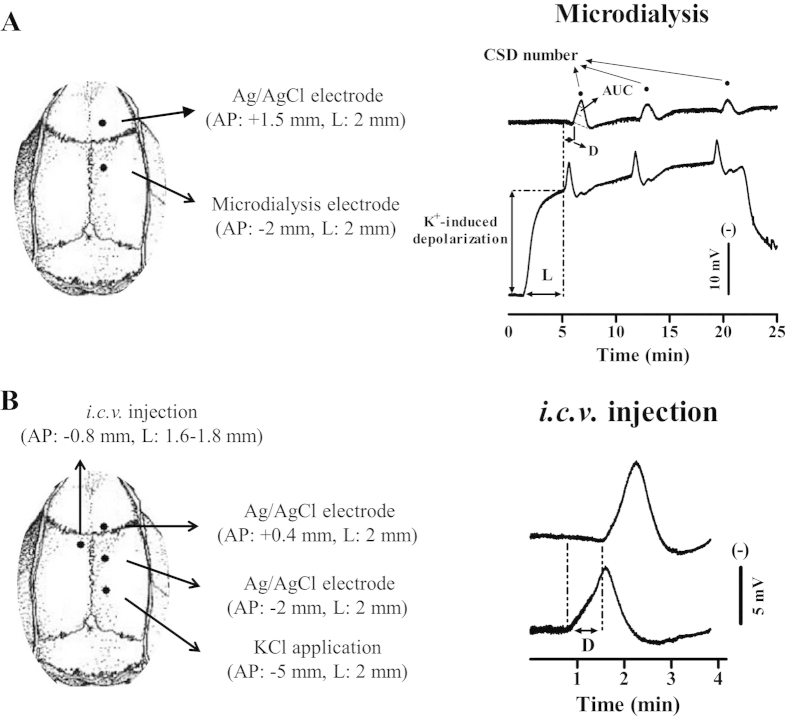
Schematic representation of electrode implantation sites (left) and representative traces in DC potential of CSD (right) induced by K^+^ through microdialysis probe (upper) and topical application (lower) in the anaesthesized rat. (**A**) In microdialysis based CSD experiment (upper), CSD wave in the genesis site was recorded by perfusion of 250 mM KCl through the microdialysis probe implanted at 2 mm posterior to bregma in the rat right cortex. CSD propagation wave was simultaneously recorded through the Ag/AgCl electrode implanted at 1.5 mm anterior to bregma. Drugs or ACSF was perfused through the microdialysis probe for pharmacology study. (**B**) In intracerebral ventricle (*i.c.v*) based experiment (lower), CSD was induced by topical application of 3 M KCl through the hole with dura intact at 5 mm posterior to bregma in the right skull. CSD propagation wave was recorded at both 2 mm posterior to and 0.4 mm anterior to bregma. The drug or ACSF was perfused into contralateral ventricle through a cannula implanted (lower left). CSD magnitude is indicated as Area under the curve (AUC, dotted line, right upper trace). CSD latency (L) and CSD number are used to assess the susceptibility of the cortex to K^+^ induced CSD. CSD propagation rate is used to assess the susceptibility of the cortex to CSD propagation as calculated by the distance dividing by the time delay (D, right).

Surprisingly, local application of NVP-AAM077 at 0.3 μM through the microdialysis probe did not alter the magnitude of CSD propagation wave and propagation rate in rats (Fig. 2B,C), which is in contrast to that was reported in chick retina, in that NVP-AAM077 was effective in suppressing CSD propagation rate and magnitude^15^. We suspected that this negative result *in vivo* may be attributed to the fact that the anterior CSD propagation site was not exposed to NVP-AAM077 when the drug was locally perfused through microdialysis probes at the CSD elicitation site (Fig. 5A). To clarify whether NVP-AAM077 was effective in CSD propagation *in vivo*, contralateral ventricle perfusion of NVP-AAM077 (total 0.3 nmol) was considered as this would allow the drug to diffuse to distant cortex by following cerebrospinal fluid flow. Indeed, it has previously been demonstrated in rats that a larger molecule, insulin, could flow to almost the whole cortex in rats within 2 hours after contralateral ventricle injection, with approximate 1/100 of the drug distributed to the contralateral cortical tissue 30 minutes after injection^26^. In our study, as expected, the 0.3 nmol of NVP-AAM077 perfused into the contralateral ventricle was found to markedly suppress both the magnitude of CSD propagation wave (Fig. 3A) and propagation rate in rats (Fig. 3B), confirming NR2A mediates CSD propagation^15^.

The importance of NR2A in CSD propagation was further supported by the action of TCN-201 (Fig. 4), which has 300-fold selectivity to NR2A compared with NR2B-containing receptors^17^. Indeed, at the medium concentration of 3 μM of the drug applied, suppressive effects on both propagation rate and magnitude of CSD propagation wave was observed in chick retina (Fig. 4). As the chick retina is devoid of blood vessels, the fact that the inhibitory effect of TCN-201 and NVP-AAM077 on CSD propagation under our study suggests an involvement of neuronal or glial mechanism under NR2A inhibition. Interestingly, this data on TCN-201 in chick retina is in contrast to a recent study employing BOLD fMRI approach, which showed that TCN-201 was ineffective in CSD BOLD response in rats^16^. The discrepancy may be attributed to different methods applied for CSD recording: BOLD fMRI^16^ that is largely based on brain-specific hemodynamic responses *versus* intrinsic optical signal that is based on neuronal activity of chick retina (Fig. 4). Whether TCN-201 could alter CSD *in vivo* using an electrophysiology approach where neuronal activities could be monitored requires further investigation. It is noted that the efficacy of TCN-201 against CSD in chick retina is >30-fold lower than that of NVP-AAM077 (Fig. 4A). This suggests that a much higher concentration of TCN-201 may be needed for sufficient binding to its receptors in future *in vivo* studies for effectiveness of the drug on CSD. Further studies using NR2A knockout animals will also help to elucidate the role of NR2A in CSD.

Elucidation of the inhibitory effects of NVP-AAM077 on CSD in *in vivo* suggests that NVP-AAM077 or such drug-alike candidates targeting NR2A may constitute a highly specific strategy with better efficacy and safety profile for treating CSD associated migraine aura, relative to non-subtype selective NMDA receptors^15^. This possibility is likely due to the following reasons: (1) NVP-AAM077 suppressed CSD in chick retina (Fig. 4) with approximately 320-fold more potency than memantine^8^, a clinically acceptable drug for treating migraines^27^. (ii) Partial inhibition of CSD by NVP-AAM077 in rats was also observed with the non-competitive NR2A subunit antagonist, memantine^8^,^9^. (iii) The rapid reversibility of NVP-AAM077 after the drug withdrawal (Figs 1, 2, 3) resulting from its competitive antagonism supports a likely better safety profile. (iv) Our latest preliminary data demonstrated that using a clinical acceptable route of administration (intravenous), pre-treatment of NVP-AAM077, at a dose of 2.4 mg/kg that preferably antagonizes NR2A-containing receptors^28^, suppressed tissue susceptibility to CSD induced by topical application of KCl (data was not shown).

### Mechanism contributing to the role of NR2A in CSD

The present data suggests activation of NR2A-containing receptors contributes to CSD genesis and propagation. There are several mechanisms which may explain the nature of CSD modulation by NVP-AAM077 and TCN-201 *in vivo* and *in vitro*. It is likely that inhibition of CSD genesis and propagation wave under NR2A inhibition is attributed to their ability to desensitize neurons (as NVP-AAM077 competitively blocks steady-state NR1/NR2A receptor currents from a cortical neuron)^29^; whereas TCN-201 accelerates NR1/NR2A receptor deactivation^30^. This notion was supported by that the inhibitory effect of NVP-AAM077 and TCN-201 on CSD propagation was observed in the chick retina, a tissue devoid of blood vessels, suggesting neuronal mechanism under NR2A inhibition. Alternatively, the suppression of CSD in response to NR2A inhibition may be based on the NR2A-mediated steady-state current permitting calcium ion influx in neurons^31^; whilst the increased calcium influx triggers and accelerates CSD in rats^32^. Finally, the role of NR2A in CSD may also be associated with its interaction with postsynaptic density protein 95 (PSD95) as NMDA receptor desensitization can be regulated by direct binding of NR2 to PDZ1-2 domains of PSD95 in neuronal and non-neuronal cells^33^. Further investigations are required to understand mechanism underlying NR2A-containing receptors in mediating CSD genesis and propagation. It is known that the NR2B containing NMDA receptor is also involved in CSD elicitation and propagation in rats using electrophysiology^8^ and BOLD fMRI^16^. Therefore, it is reasonable to propose that CSD genesis and propagation requires both NR2A and NR2B activation. Further synergistic effect on CSD under both NR2A and NR2B inhibition *in vivo* remains to be examined.

In summary, our data provides strong evidence that NR2A subunit contributes to CSD genesis and propagation, suggesting drugs selectively antagonizing NR2A-containing receptors might constitute a highly specific strategy treating CSD associated migraine with a likely better safety profile.

## Methods

All animal procedures were approved by the Ethical Review Panels of Soochow University and performed in accordance with the associated guidelines. All efforts were made to minimize animal suffering to reduce the number of animals used.

### *In vivo* CSD in rats with electrophysiology

#### Animal use and anaesthesia

Adult, male Sprague Dawley rats (*n* = 30, 317 ± 40 g, mean ± SD, Shanghai SLAC Laboratory Animal Corporation Ltd) were housed with food and water available *ad libitum*. Rats were anaesthetised with isoflurane (5% for induction, 2.5–3.5% during the surgery, 1–1.5% for maintenance) in O_2_:N_2_O (1:2), with the animal breathing spontaneously. The depth of anaesthesia was monitored and adjusted through careful examination of the electroencephalogram (EEG) signal and through observation of the animal. Rectal temperature of animals was maintained at 37 °C. CSD induction was carried out one hour after the surgery for tissue stabilization.

#### Microdialysis-based CSD experiment

To record CSD genesis and propagation waves, two burr holes were drilled on the right parietal bone (Fig. 5A). (i) Microdialysis probes incorporating an electrode (ME-H1, Applied Neuroscience, London) were implanted through the posterior burr hole (coordinates: 2 mm posterior and 2 mm lateral to bregma, 1.4–1.5 mm deep from the cortical surface) for recording of CSD genesis waves; (ii) Ag/AgCl electrodes (0.1 mm i.d, Applied Neuroscience, London) were implanted into the ipsilateral cortex through the anterior burr hole (coordinates: 1.5 mm anterior and 2 mm lateral to bregma, 0.9 mm deep from the cortical surface) for recording of CSD propagation waves; separate Ag/AgCl reference electrodes were placed under the scalp of rats. EEG and Direct Current (DC) potential were derived from the potential between microdialysis probes or Ag/AgCl electrodes and reference electrodes. The microdialysis probe was perfused with artificial cerebrospinal fluid (ACSF) at 1 μl/minute with a syringe pump (CMA100, CMA/Microdialysis, Solna, Sweden). For CSD elicitation, a medium containing 250 mM K^+^ (K^+^-medium, composition in mM: 2.5 NaCl, 250 KCl, 1.18 MgCl_2_, 1.26 CaCl_2_; pH 7.3 adjusted with 1 M NaOH, not buffered) was used; It is noted that a higher concentration of K^+^ at 250 mM was required to induce CSD compared to 160 mM employed when halothane was used as the anaesthetic^34^. This difference in K^+^ requirement could be due to the anaesthetics (isoflurane *vs* halothane) having different effects on CSD^18^, but this effect can be ignored as all experiments were carried out under the same anesthetic condition.

One hour after the surgery, drugs or ACSF were perfused through the microdialysis probe at 1 μl/minute. In order to examine the effects of NVP-AAM07 on CSD genesis and propagation, the following series of experiments were carried out. The known non-competitive NMDA receptor antagonist, MK801, was used as the reference. These series are: (i) ACSF (Control, *n* = 6); (ii) MK801 (Sigma-Aldrich, Dorset, UK, *n* = 6); (iii) NVP-AAM077 (*n* = 6), which was synthesized following the procedures described in the literature^35^ and further purified by HPLC. It should be noted that the microdialysis probe used in this study has ~10% recovery rate, *i.e.* the ratio of the drug penetration through the semi-permeable membrane of the probe into the cortex relative to its original concentration. An estimation of 0.3 μM of NVP-AAM077 was expected to diffuse into the cortex surrounding the microdialysis probe implantation site when the maximum concentration at 3 μM of the drug was applied, at which, the drug is preferably selective for NR2A subunit^36^.

Five consecutive CSD episodes were elicited in each experiment by perfusion of K^+^-medium through microdialysis probes for 20 minutes followed by 40 minutes ACSF perfusion to allow tissue recovery. These five episodes were: (i) initial ACSF control; (ii) low concentration of the drug or ACSF; (iii) medium concentration of the drug or ACSF; (vi) high concentration of the drug or ACSF; (v) post-treatment with ACSF solution. The drug at each concentration (0.3, 1, 3 μM for NVP-AAM077 or 3, 10, 30 μM for MK801) was perfused through microdialysis probes for 20 minutes before and during high K^+^-medium perfusion for the 2^nd^, 3^rd^ and 4^th^ CSD episodes.

#### Intracerebroventricular injection experiment

To ensure NVP-AAM077 diffusion to a wider cortical region for investigation of CSD propagation under NR2A inhibition, a separate set of *in vivo* experiment by perfusion of NVP-AAM077 into the left lateral ventricle was designed. Three burr holes were drilled in the right and left parietal bones (Fig. 5B) for the following purposes: (i) one burr hole (1 mm i.d) with dura intact was drilled in the right parietal bone (coordinates: 5 mm posterior and 2 mm lateral to bregma) for inducing CSD by topical application of 1 μl of 3 M KCl for 1 minute; (ii and iii) two burr holes were drilled on the ipsilateral area (coordinates: 2 mm posterior and 0.4 mm anterior to bregma respectively, 2 mm lateral) and Ag/AgCl recording electrodes were implanted into the cortex (0.9 mm deep from the cortical surface) through these burr holes for recording CSD propagation waves. The DC potential was derived between the Ag/AgCl electrodes and a reference electrode placed under the scalp; (vi) A stainless steel cannula (inner diameter: 0.38 mm, RWD Life Science, Shenzhen, China) was implanted into the contralateral ventricle (coordinates: 0.8 mm posterior and 1.6–1.8 mm lateral to bregma, 3.5 mm deep from the cortical surface) and held in place by acrylic dental cement for the drug or ACSF perfusion. These burr holes were kept moisture with ACSF throughout the experiment.

After one hour stabilization post-surgery, two CSD episodes were induced by topical application of 1 μ1 of KCl at 3 M with 40 minute interval. NVP-AAM077 (0.3 nmol, *n* = 6) or ACSF (Control, *n* = 6) was perfused at 1 μl/minute for 10 minutes using a syringe pump (CMA100, CMA/Microdialysis, Solna, Sweden) into the contralateral ventricle immediately after the 1^st^ CSD wave of each episode was completed.

#### Recording of EEG and extracellular direct current potential

EEG and DC signal recording was as reported previously^34^. Briefly, EEG and DC signals were amplified using an AC/DC pre-amplifier (NL834, Neurolog System, Digitimer Ltd., Welwyn Garden City, UK). All the recorded variables were continuously digitised, displayed and recorded by a computer using Labview 11.0 (NI Instruments) via an analogue/digital-converter (USB6009, NI Instruments). In the microdialysis-based experiment, CSD at the induction site was recognized as a transient, negative shift superimposed on the sustained shift resulting from the imposed high extracellular K^+^ concentration (Fig. 5A, lower trace). CSD propagation wave was recognized by a transient, negative shift but in the absence of the sustained depolarization shift (Fig. 5A, upper trace and 5B).

#### *In vivo* data presentation and statistical analysis

Details on CSD number, latency and magnitude in rats were quantified as that described previously (Wang *et al.*^34^). Labview program was used to determine (i) CSD number in each episode; (ii) Latency (minute), the time required to elicit the 1^st^ CSD wave from the start of depolarization induced by high K^+^-medium. In the case of the CSD wave being completely abolished by drug(s), the latency was counted as 20 minutes. CSD number and latency were used to reflect cortex susceptibility to CSD; (iii) Area under the curve (AUC, mV × minute) of CSD waves was used to reflect CSD magnitude. The magnitude of each CSD wave in each CSD episode was averaged for data comparison. Values were counted as zero when CSD waves were abolished; (vi) CSD propagation rate (mm/minute). The velocity of each CSD wave in each CSD episode was averaged for data comparison. All values were given in mean ± SEM. CSD number and latency was analysed using one-way ANOVA with subsequent Bonferonni test for significance between drugs and control group. CSD magnitude and propagation rate were analysed using unpaired t-test between the drug and control group. Paired t-test was used to test the significance of the effect of drug removal.

### *In vitro* CSD in chick retina with intrinsic optical imaging

#### CSD induction in chick retina and intrinsic optical imaging

Twenty six male chicks (purchased at 1 day old, WuXi Yangzichang Ltd, Wuxi, China) were housed for at least a week before use (aged 8–28 days). As described previously^37^, posterior eyeball was positioned in a chamber, which was perfused at 0.5 ml/minute with Ringer’s solution. The tissue was stabilized for at least 30 minutes before CSD induction and temperature kept constant at 32 °C.

Retinal CSD was induced as previously described^15^,^37^. Briefly, ten repeated CSD episodes were induced by ejection of 1 μl of KCl (0.1 μM) at the eyeball with 20-minute interval for tissue recovery. The retina was illuminated for 25 ms at 1 Hz using a high-power LED spotlight (625 nm peak wavelength, SLS-0307-A, Mightex; Pleasanton, CA, USA) and the illumination was driven by a computer-controlled power supply (Sirius LED controller, SLC-SA04-U; Mightex, Pleasanton, USA). The reflected light was simultaneously recorded with a monochrome camera (QIC-F-M-12, Media Cybernetics, Marlow, UK). Image sequences were taken at 1 Hz over a 3-minute period, started as CSD was elicited. Camera exposure and illumination were synchronized using the same external trigger (TG5011, TTi, UK). Image Pro Plus software (IPP 7.0; Media Cybernetics UK, China) was used for image acquisition, storage and analysis.

#### *In vitro* experimental design for drug testing

Four groups were designed and the concentration range of each drug was carefully selected to ensure that each drug tested was preferably selective for NR2A subunit: (i) TCN-201 (Tocris, Bristol, UK) at 1, 3 and 9 μM (*n* = 6); (ii) NVP-AAM077, at 0.03, 0.1 and 0.3 μM (*n* = 6) as the positive control; (iii) DMSO (Sigma-Aldrich, Dorset, UK) at 0.001%, 0.03% and 0.1% as the TCN-201 vehicle group in respective order (*n* = 8); It should be noted the maximum concentration of DMSO without affecting CSD in chick retina was 0.1% DMSO per our preliminary experiment; (vi) Ringer’s solution as the control (*n* = 6) for NVP-AAM077 group.

For each group tested, ten CSD episodes were induced in each experiment, with two separate CSDs for each of the different and consecutive tests: (i) initial Ringer’s control; (ii) low concentration of drug or vehicle; (iii) medium concentration of drug or vehicle; (vi) high concentration of drug or vehicle; (v) post-treatment with Ringer’s control (*i.e.* drug removal). For each test sequence, the perfusion medium was changed immediately after the end of the 2^nd^, 4^th^, 6^th^, and 8^th^ CSD recording when required, so that the preparation was adequately perfused with the proper drug, vehicle, or Ringer’s medium for the subsequent test.

#### *In vitro* data presentation and statistical analysis

As reported previously (Wang *et al.*^15^), for each image sequence, an area of interest (AOI) parallel to the CSD wave front was delineated manually. For each image within the sequence, the gray levels of the pixels constituting the AOI were averaged and plotted against the time as an indicator to characterize CSD. For each CSD wave, the area under the curve (AUC, gray levels × minute) of the transient cellular depolarization was calculated and used as an index of the magnitude of propagating CSD. For each CSD wave, propagation rate was also calculated to reflect the degree of tissue excitability. The calculated values within each different test were averaged and all corresponding data were given as mean ± SD in percentages of their respective baselines. Kruskal–Wallis test was used with subsequent Dunn’s test for comparison of the magnitude and propagation rate of RSD between the drug and respective control group. Wilcoxon matched pairs test was used to test the significance of the difference for the last two tests with each drug (*i.e.* effect of drug removal).

## Additional Information

**How to cite this article**: Bu, F. *et al.* NR2A contributes to genesis and propagation of cortical spreading depression in rats. *Sci. Rep.* **6**, 23576; doi: 10.1038/srep23576 (2016).
